# A cohort study of a general surgery electronic consultation system: safety implications and impact on surgical yield

**DOI:** 10.1186/s12913-017-2375-0

**Published:** 2017-06-23

**Authors:** Jesus G. Ulloa, Marika D. Russell, Alice Hm Chen, Delphine S. Tuot

**Affiliations:** 1 Veterans Affairs/Robert Wood Johnson Foundation, Clinical Scholars Program, University of California Los Angeles, Los Angeles, CA 90095 USA; 2 Renal Center at Zuckerberg San Francisco General Hospital and Trauma Center, 1001 Potrero Avenue. Bldg 100, Room 342, San Francisco, CA 94110 USA; 3 Department of Otolaryngology, University of California, San Francisco, CA 94143 USA; 4 Department of Medicine, University of California, San Francisco, CA 94143 USA

**Keywords:** Electronic consultation, Surgical yield, Ambulatory safety, Patient-centered medical neighborhood, Health system redesign

## Abstract

**Background:**

Electronic consultation (eConsult) systems have enhanced access to specialty expertise and enhanced care coordination among primary care and specialty care providers, while maintaining high primary care provider (PCP), specialist and patient satisfaction. Little is known about their impact on the efficiency of specialty care delivery, in particular surgical yield (percent of ambulatory visits resulting in a scheduled surgical case).

**Methods:**

Retrospective cohort of a random selection of 150 electronic consults from PCPs to a safety-net general surgery clinic for the three most common general surgery procedures (herniorrhaphy, cholecystectomy, anorectal procedures) in 2014. Electronic consultation requests were reviewed for the presence/absence of consult domains: symptom acuity/severity, diagnostic evaluation, concurrent medical conditions, and attempted diagnosis. Logic regression was used to examine the association between completeness of consult requests and scheduling an ambulatory clinic visit. Surgical yield was also calculated, as was the percentage of patients requiring unanticipated healthcare visits.

**Results:**

In 2014, 1743 electronic consultations were submitted to general surgery. Among the 150 abstracted, the presence of consult domains ranged from 49% to 99%. Consult completeness was not associated with greater likelihood of scheduling an ambulatory visit. Seventy-six percent of consult requests (114/150) were scheduled for a clinic appointment and surgical yield was 46%; without an eConsult system, surgical yield would have been 35% (*p*=0.07). Among patients not scheduled for a clinic visit (*n*=36), 4 had related unanticipated emergency department visits.

**Conclusion:**

Econsult systems can be used to safely optimize the surgical yield of a safety-net general surgery service.

## Background

Implementation of electronic consultation systems (eConsult) systems has been pursued across varied disciplines to enhance access to specialty expertise [1]. eConsult systems are technology-enabled applications that allow primary care providers (PCP) to electronically submit requests for specialty expertise. These systems permit referral management and tracking by primary care teams, allow for specialist triage to ensure that patients with acute issues are seen in a timely fashion, and when appropriate, encourage virtual co-management between primary care and specialty care with iterative bidirectional communication [2].

Studies of eConsult systems have found decreased evaluation time for workup of medical conditions (i.e., hematuria), improved PCP access to specialty expertise from timely eConsult responses, and shorter wait times for in-person specialty care appointments, while maintaining high PCP, specialist and patient satisfaction [3–10]. There is limited literature on the impact of eConsult systems upon the efficiency of specialty care delivery. One measure of such efficiency among surgical services is surgical yield, defined as the percentage of ambulatory surgical consultations leading to scheduling of an operative intervention [11].

Measuring the impact of eConsult systems on surgical yield, along with its determinants, may provide additional data to encourage their adoption by public healthcare delivery systems that traditionally have had poor surgical access [3, 12, 13], suboptimal inter-provider communication and care coordination, inappropriate referrals [14, 15], and high wait times for ambulatory clinic visits [16, 17]. The consequences of poor access to ambulatory specialty services within public health care systems, in particular surgical, include increased risk of requiring urgent or emergent surgery, longer lengths of hospitalizations, and decreased likelihood of receiving follow-up care [18–20].

Our specific objectives with this study were three-fold: (1) to evaluate the impact of an eConsult system on surgical yield for a safety-net general surgery service; (2) to examine whether the completeness of electronic consultation requests was associated with the decision to schedule or not schedule an ambulatory general surgery clinic visit; and (3) to assess the potential safety implications related to the triage function of a general surgery eConsult program, which could serve as a balancing measure to surgical yield. We hypothesized that the eConsult system would safely increase surgical yield and that completeness of electronic consultative requests would be positively associated with scheduling of an ambulatory care appointment.

## Methods

### Study setting

Zuckerberg San Francisco General Hospital and Trauma Center (ZSFG) is the acute care hospital for the San Francisco Health Network (SFHN), the City’s publicly funded integrated delivery system. Serving over 100,000 patients annually, ZSFG also provides a full range of ambulatory specialty services. Referrals to specialty care come from a network of 14 SFHN primary care clinics as well as from a consortium of ten affiliated community clinics that rely on ZSFG for elective specialty, diagnostic and inpatient care. Since 2009, all referrals to ZSFG specialty services are made through an integrated electronic referral and consultation program developed by University of California physicians at ZSFG, known as eReferral [21].

The general surgery clinic at ZSFG is staffed by nine general surgeons, is the only general surgery clinic for the SFHN, and experiences a large supply-demand mismatch consistent with other public healthcare specialty services. Referring providers use eReferral to electronically submit all consultation requests to specialty care, including general surgery. All requests for specialty expertise submitted by providers to the general surgery service are reviewed by one general surgery clinician who is remunerated to perform this service prior to scheduling an outpatient surgical visit. The general surgery reviewer may reply to the referring provider (most often a PCP) requesting additional pertinent history, advise diagnostic evaluation (i.e. imaging, blood work, etc), suggest consulting a different specialty service (i.e. pediatric surgery for infant inguinal hernia), or recommend medical treatment (i.e. fiber supplementation for hemorrhoids). After iterative asynchronous electronic communication between providers, the reviewer may schedule the patient for in-person evaluation, or advise ongoing treatment that can be delivered in the patient’s medical home without need for a surgery clinic appointment. Prior to implementation of eReferral, all requests for surgical expertise were made by fax or telephone and all patients were scheduled for a general surgery clinic visit on a first-come first-serve basis without any pre-visit communication among providers.

### Study design

We retrospectively reviewed electronic consultation requests submitted to the adult general surgery clinic for the 2014 calendar year and abstracted variables including: date consult was submitted, patient name, patient medical record number, reason for consult text, note history containing communication between the referring provider and general surgery reviewer, primary care provider type, primary care clinic, and date of outpatient surgery appointment, if applicable. Consults placed by primary care providers (family medicine or internal medicine) were included; referrals from other referring provider types (i.e., gastroenterologists, pediatricians) were excluded.

Consult requests were categorized into three groups based on the presence of keywords commonly used when describing diagnoses of (1) fascial defects (hernia, abdomen, pain, bulge, incarcerated, groin, recurrence), (2) biliary tract pathology (gallbladder, gallstones, biliary colic, right upper quadrant pain, jaundice, cholelithiasis, cholangitis, elevated bilirubin, pancreatitis) and (3) anorectal diseases (hemorrhoid, fissure, anal pain, blood per rectum, mass, abscess, fistula, condyloma, fulguration, recurrence, polyp). These three categories were chosen because they correspond to the most common elective surgeries performed by the ZSFG General Surgery Department: herniorrhaphy, cholecystectomy and anorectal procedures.

### Consultation completeness assessment tool

**Fig. 1:**
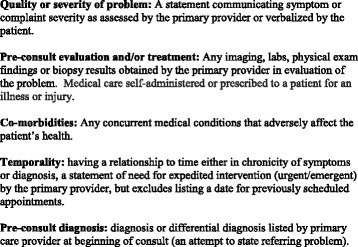
Consultation completeness assessment tool

The study authors iteratively defined the domains of a consultation request, based on their prior work examining the quality of communication among referring and specialty providers [2, 5, 22]. Domains included symptom acuity and severity, diagnostic evaluation, concurrent medical conditions, and attempted diagnosis. To ensure high inter-rater reliability, two study members independently coded 15 random consult excerpts (30 total) in two separate rating rounds. Disagreements were discussed and mediated by a third study member to reach consensus of descriptor definitions. Inter-rater reliability for each domain was measured using Cohen’s kappa and ranged from 0.43 to 0.76, indicating moderate to substantial agreement in application of our quality descriptors. Domains in the final evaluation tool included: quality/severity of problem, pre-consult evaluation and/or treatment, patient co-morbidities, temporality and pre-consult diagnosis (Fig. 1).

### Data analysis

Fifty randomly chosen consults from each surgical referral category (fascial defects, biliary tract pathology, anorectal diseases) were abstracted for our final study cohort. This ensured a broad representation of consultation requests, despite not representing a random sample of all surgery consults. With a sample size of 150, we anticipated having 80% power to identify an increase in surgical yield of 30%, assuming a baseline surgical yield of 30% based on historical data. We calculated the distribution of consultation requests by referring provider, referring primary care clinic, and referral domain variables. The general surgery operative schedule was reviewed from January 1, 2014 through December 31, 2015 and compared to the final study cohort by patient name and medical record number to identify referred patients who were scheduled for surgery. Consults were subsequently de-identified of patient, provider, and clinic specific information and assessed for completeness, using the aforementioned tool.

Bivariate associations between scheduling of an ambulatory clinic visit and consult domains (quality or severity of problem, pre-consult evaluation, co-morbidities, temporality, pre-consult diagnosis) were measured using Pearson’s chi square. The odds of requiring iterative communication by the specialist and being scheduled for an ambulatory care clinic were modeled using multivariate logistic regression that included all the presence/absence of consult domains (binary variables), referring provider type (binary variable) and primary care clinic type (binary variable). Surgical yield, defined as the percent of all ambulatory consultations that resulted in a scheduled surgical case, was calculated.

To assess for potential harms in patient care that could occur with overzealous patient triage to maximize high surgical yield, we reviewed unanticipated health care utilization (urgent care and emergency department visits, hospital admissions, and surgical cases) through December 31 2015 for patients who were not scheduled for an ambulatory general surgery visit based on information contained in the electronic consultation request, or for those patients who were scheduled but required more urgent admission and inpatient surgery while awaiting their general surgery clinic visit. Analyses were performed using Stata 13.0 (College Station, TX). This study was approved by the Institutional Review Board of the University of California San Francisco (study number: 15–15,794).

## Results

### Use of the eConsult system and surgical yield

There were 1743 electronic consultations submitted to general surgery during the 2014 calendar year. Of these, we identified 607 for fascial defects, 241 for biliary diseases, and 264 for anorectal diseases. Among the 50 randomly selected consultation requests from each category (150 in total), the referring provider was a physician in 65% of consults (Table 1).

**Table 1:** Consult characteristics; *N*=150

Referring provider	
Nurse Practioner/Physician Assistant	53 (35%)
Physician	97 (65%)
Referring primary care clinic^a^	
SFDPH	51 (34%)
UCSF	59 (39%)
Independent	40 (27%)
Frequency of measured domains	
Quality or severity of problem	132 (88%)
Pre-consult evaluation and or treatment	140 (93%)
Co-morbidities	74 (49%)
Temporality	99 (66%)
Pre-consult diagnosis	148 (99%)
Clinic appointment scheduled	114 (76%)
Surgical case scheduled	53 (35%)
Surgical yield	53/114 (46%)

^a^ *SFDPH* San Francisco Department of Public Health, ^a^ *UCSF* University of California San Francisco

Overall, 28% of consultation requests were not scheduled for a general surgery clinic appointment. Similarly, among the 150 consultation requests reviewed, nearly one quarter (*n*=36, 24%) were not scheduled for an in person clinic appointment. Reasons for not scheduling an in-person visit included the following: patient was recommended for referral to another specialty service (*N*=9), specialty reviewer requested that additional workup be completed by PCP (*N*=11), an in-person ambulatory surgical visit was deemed as not indicated based on the iterative electronic consultative communication and patients remained in primary care (*N*=8), conservative treatment was advised with symptom re-evaluation by PCP and resubmission of the consultation request as needed (*N*=7), and there was incomplete electronic closure of the consultative communication by the PCP (*N*=1). The consultative exchange resulted in 76% of consultation requests scheduled for a general surgery clinic appointment, either immediately or after iterative communication between the PCP and specialist reviewer. Forty-six percent of patients scheduled for an in-person visit (*n*= 53/114) were scheduled for a subsequent surgical intervention, representing surgical yield. Without an electronic consult system, all 150 requests for general surgery expertise would have required an ambulatory clinic visit prior to scheduling a surgery, with a surgical yield of 35% (*p*=0.07 for difference in surgical yield).

### Impact of the completeness of consultation requests

The frequency of consult domains included in the initial consultation request varied from 49% to 99% (Table 1). In general, completeness of the consultation request was not associated with the scheduling of a clinic appointment for surgical evaluation (Table 2). The only consult domain that was associated with scheduling of a clinic appointment for surgical evaluation was inclusion of a pre-consult diagnosis (*p*=0.01). In a multivariate model that also included provider and clinic type, none of the domains were associated with increased odds of an ambulatory clinic visit being scheduled (Table 2).

**Table 2:** Odds of surgical consult request scheduled for appointment

Covariate	Univariate Odds ratio	Adjusted Odds Ratio
Quality or severity of problem	0.50 (0.19–1.35)	0.48 (0.17–1.36)
Preconsult evaluation and or treatment	1.85 (0.47–11.21)	2.38 (0.45–12.48)
Co-morbidities	0.16 (0.31–1.21)	0.63 (0.31–1.28)
Temporality	0.72 (0.44–1.78)	0.86 (0.41–1.81)
Referring provider		
Nurse Practitioner/Physician Assistant	reference	
Physician	0.58 (0.29–1.16)	0.66 (0.32–1.35)
Primary care clinic^a^		
SFDPH	reference	
UCSF	0.95 (0.43–2.11)	1.09 (0.46–2.57)
Independent	1.47 (0.37–3.48)	1.25 (0.49–3.20)

^a^ *SFDPH* San Francisco Department of Public Health, ^a^ *UCSF* University of California San Francisco

### Safety considerations

Among the 36 consultation requests that were not scheduled for a general surgery clinic appointment via the electronic consultation and referral  system, 4 patients required urgent care/emergency room evaluation for complaints related to the content of their consult request. Of those patients, 2 required admission to the hospital. In both scenarios, the surgical reviewer had recommended additional diagnostic procedures (i.e., imaging, colonoscopy) prior to scheduling an ambulatory clinic visit, and the patients presented to the emergency department prior to completing the recommended workup, prompting hospital admission, inpatient completion of the workup, and definitive surgical management during the hospitalization. Among the 2 patients that did not require admission to the hospital, one presented to the emergency department prior to completing the diagnostic workup recommended by the general surgery reviewer and was scheduled for an elective surgery without an ambulatory general surgery clinic visit after completion of the workup. The other patient presented to the emergency department on the same day that his PCP submitted the consultation request to general surgery and was recommended non-surgical management. Additionally, among the patients who were scheduled for an outpatient surgical consultation, there were 4 patients who required emergency room evaluation before their scheduled outpatient visit. Each of these scenarios resulted in an eventual surgical procedure.

## Discussion

This study has two key findings that are pertinent to the adoption of eConsult platforms in health care delivery systems. First, we demonstrated that an integrated electronic consultation and referral system, in which all referrals are submitted electronically and reviewed by a general surgery clinician, can safely increase the surgical yield of an ambulatory general surgery service. Second, we determined that referral completeness by PCPs for common general surgery consultation requests is variable and is not associated with odds of a patient being scheduled for an ambulatory visit.

Care coordination is one of the tenets of the American College of Physicians’ patient centered medical home-neighborhood (PCMH-N) and the Centers for Medicare and Medicaid Services’ Accountable Care Organization (ACO) models of care delivery. Both of these models encourage shared responsibility and accountability for the efficient delivery of high-quality medical and surgical services [23–25]. ACOs additionally aim to enhance value through aligned provider and system incentives [26]. To date, both of these models have focused on chronic care delivery. Little effort has been directed to evaluating surgical referral patterns or including surgeons and surgery practices in ACOs [27].

Several metrics pertinent to surgical care delivery are similar to those that have already been proposed for chronic medical care delivery, including those that measure care coordination, frequency and quality of inter-provider communication, and patient satisfaction. Value metrics for surgical services may be different than their medical counterparts, however. We propose surgical yield as one such metric. There is no accepted gold standard value for surgical yield. High surgical yield implies that patients who would most benefit from a surgical intervention are evaluated by surgical clinicians during limited ambulatory clinic visit times and scheduled for surgery, and that patients who would not benefit from a surgical intervention are co-managed virtually and remain in primary care. Low surgical yield implies that all referred patients are evaluated in an ambulatory surgical clinic, including those with low likelihood of benefiting from a surgical procedure. It is important to note that a surgical yield of 100% may not be ideal, as there are cases for which non-operative in-person co-management between PCPs and surgeons is superior to operative management and perhaps virtual co-management (i.e. patient with advanced cirrhosis and an umbilical hernia). Indeed, very high surgical yield may be associated with the improper triage of patients back to primary care who would in fact benefit from an in-person surgical evaluation. The goal for high-functioning health systems, thus, is to safely optimize surgical yield, with the highest percentage of ambulatory general surgery patients receiving indicated elective surgery without any need for unanticipated emergent or urgent interventions.

Determinants of surgical yield are not clear, but may include appropriateness of referrals to a surgical service, completeness of information communicated between referring providers and surgeons for triaging purposes, availability of ambulatory and operative services, and demographic and clinical characteristics of referred patients. In this study, we did not find that referral completeness was associated with greater odds of being scheduled for an ambulatory clinic visit or surgical yield. Our findings confirm prior work demonstrating PCPs vary in their communication of necessary information to their specialist colleagues [28], and suggest that results of imaging studies or other objective data may be more important for surgical triage than the content of a referral request.

eConsult systems can be leveraged to improve the completeness of specialty referrals, however. In our study, common reasons for not scheduling a surgical clinic appointment included a request for more diagnostic evaluation and recommendation for trials of conservative non-surgical treatment as an initial measure. This type of case-based education may indirectly teach PCPs important diagnostic and therapeutic content to include in consultation requests [29]. More directly, referral guidelines and consult templates embedded within eConsult platforms that force responses to pre-populated options (i.e. comorbidities, symptom severity, temporality, etc), can indicate to referring providers the necessary content for complete consultation requests. Interestingly, referral guidelines have demonstrated improved appropriateness of pre-consult diagnostic evaluation for general surgery [30] and for pediatric orthopedic clinics [14], but they have not been associated with improved health outcomes among referred patients [30]. Additionally, integration of eConsult programs with electronic medical record systems could enhance the completeness of consultation requests by automatically including patient comorbidities and components of the pre-consult evaluation (imaging or blood work).

The safety implications of eConsult programs that allow for triage have not been extensively studied and remain an area of inquiry for this field [31]. However, our findings suggest that an integrated electronic consultation and referral system that relies on collaborative communication among PCPs and specialist providers, can help health systems safely optimize their surgical yield. We found few instances of unplanned healthcare utilization related to triage facilitated by the eConsult program. Only 8 patients (5.3%) required unscheduled emergency department care. Four of these patients presented to the emergency department before their scheduled ambulatory general surgery clinic evaluation, reflective of the lengthy wait times associated with the limited capacity of ambulatory surgical services in safety-net healthcare delivery settings. Among the 4 patients who were not scheduled for an outpatient visit, one patient presented to the emergency department on the same day that the PCP submitted the general surgery consultation request, potentially indicating poor communication between the PCP and the patient. Three patients, however, presented to the emergency department while undergoing the additional workup recommended by the surgery reviewer to guide decision making, representing potential harm related to electronic consultation systems for general surgery. Without the eConsult program, these 3 patients would likely have been scheduled for an ambulatory surgery clinic evaluation. Given the lengthy wait times for in-person ambulatory clinic visits in public healthcare delivery systems, it is not clear whether these clinic evaluations would have been soon enough to avoid emergency department visits or whether they would have led to expedited surgical management.

Telephone consultations could similarly allow health delivery systems to safely increase their surgical yield. However, integrated electronic consultation and referral systems have several advantages compared to an immediate phone call exchange for non-urgent medical situations. First, surgical providers are often not available during normal business hours for telephone discussions given operative responsibilities. Specialists have cited the asynchronous nature of electronic exchange as a great strength of electronic consultation platforms [32]. Secondly, the electronic consult system also permits a review of completed preoperative surgical evaluation, including review of imaging studies when appropriate, and results in written recommendations by a specialist that are documented in a patient’s chart. This documentation minimizes any confusion that may result from a hurried telephone encounter. Additionally, PCPs may refer to the documented recommendations at a later time and apply them to other, similar patient encounters.

Of interest, other systems have tried to enhance surgical care delivery by eliminating the need for pre-operative ambulatory care surgical visits rather than focusing on surgical yield. In such systems, PCPs submit referral requests that include demographic, medical history, and physical exam data. A surgeon-led telephone triage subsequently ensues, consisting of additional medical history, a validated symptom assessment tool, and informed consent for the procedure with a patient knowledge questionnaire to ensure understanding. On the day of surgery, patients discuss the procedure again with their surgeon and provide written informed consent. In the United Kingdom, for example, such services for laparoscopic hernia repairs and cholecystectomies have been safely piloted in the past few years. In one pilot study, among 517 patients referred with hernias, approximately 2% were converted to a pre-operative in-person ambulatory care surgical visit after telephone consultation and 0.8% experienced a same-day surgery cancellation rate [33]. Among 166 patients referred for cholecystectomy, 8% were converted to a pre-operative surgical visit, 5% presented to the emergency room while awaiting surgery, and there were no same-say surgery cancellations [34]. While comparative studies with usual care referral practices are needed, this approach appears to be another promising way to safely increase the efficiency of specialty care delivery, perhaps in conjunction with optimization of surgical yield from ambulatory care surgery visits.

The results of our study should be considered alongside its limitations. We lacked a standardized method to evaluate referral completeness, though our internally developed rating tool demonstrated moderate to substantial inter-rater reliability. We could not account for emergency department visits, hospitalizations or urgent surgical interventions that occurred in other hospital systems, though all patients in this study rely exclusively on ZSFG for non-urgent specialty and diagnostic care. Nonetheless, this limitation could minimize the safety implications of a general surgery electronic consultation program. Additionally, surgical yield may be sensitive to lag resulting from a selected cut off date by which a surgical procedure occurred. We felt that our selection of a two-year time period for scheduling a surgical cases was robust to establish the range of experiences with our electronic consult system. Also, since 2009, providers have used the electronic consultation system to request ambulatory surgery expertise; as such, this study did not have a contemporary comparator group. However, prior to eReferral implementation, all patients would have been scheduled for an in-person general surgery clinic visit, allowing us to calculate a hypothetical surgical yield if no electronic consultation system was in place. It is possible that the presence of an electronic consultation system lowered the PCP threshold to submit an electronic consultation. However, implementation studies of other eConsult systems have documented that the presence of an electronic consultation system has not induced demand [35, 36], lending credibility to our assumption. Finally, results of our study are pertinent to one academic public healthcare delivery system and may not generalize to other health systems currently implementing electronic consult systems.

## Conclusion

Electronic consultation systems are an important and evolving method for increasing access to specialty services and overall provision of care within the framework of a PCMH-N or ACO. Our findings suggest that implementation of an integrated electronic referral and consultation system in which every consultation request is reviewed by a surgery clinician with subsequent communication between the referring and specialty provider, can safely optimize the surgical yield of an ambulatory general surgery service. Though further study and analyses are needed on the potential enhancements of electronic consultation systems, our experience may encourage adoption of similar systems by other healthcare delivery systems that still experience inefficient delivery of surgical care.

## Abbreviations

ACO: Accountable care organization
eCR: Electronic referral and consultation
EMR: Electronic medical record
PCMH-N: Patient centered medical home – neighborhood
PCP: Primary care provider
ZSFG: San Francisco General Hospital and Trauma Center
